# The response of glyphosate-resistant and glyphosate-susceptible biotypes of *Echinochloa colona* to carbon dioxide, soil moisture and glyphosate

**DOI:** 10.1038/s41598-019-57307-9

**Published:** 2020-01-15

**Authors:** Mahboobeh Mollaee, Ahmadreza Mobli, Bhagirath Singh Chauhan

**Affiliations:** 1 Department of Agrotechnology, Faculty of Agriculture, Ferdowsi University of Mashhad, Mashhad, 9177948974 Iran; 2 The Centre for Crop Science, Queensland Alliance for Agriculture and Food Innovation (QAAFI), The University of Queensland, Gatton, Queensland 4343 Australia

**Keywords:** Plant ecology, Abiotic

## Abstract

Physiological and growth responses of two Australian *Echinochloa colona* biotypes (glyphosate-resistant and susceptible, produced from a single population) to different concentrations of carbon dioxide (CO_2_) (ambient ~450 ppm and elevated ~750 ppm) and soil moisture (well-watered and water-stressed) were analyzed. Elevated CO_2_ and well-watered conditions resulted in *E. colona* plants with greater biomass, height and numbers of tillers and leaves in both biotypes; however, no significant response was observed for seed production or the amount of photosynthesis pigments with increasing CO_2_ at both soil moisture levels. In addition, water availability was more influential for growth than CO_2_ concentration. The mean shoot biomass of the susceptible biotype under elevated CO_2_ and well-watered conditions was significantly greater than the resistant biotype. Although the susceptible biotype showed more vegetative and reproductive growth than the resistant biotype, no significant difference was observed for seed production between the biotypes in the water-stressed condition. In a second experiment, different doses of glyphosate (0, 180, 360, 720 and 1440 g a.e ha^−1^) were applied to both biotypes grown at two soil moisture levels (well-watered and water-stressed). In the water-stressed condition, glyphosate efficacy was decreased in both biotypes. The resistant biotype in the well-watered condition had only 19% survival at 1440 g ha^−1^ glyphosate (double the recommended rate), but this value increased in the water-stressed condition by 62%. Our study suggests that future climate change can affect the physiological and growth processes of weeds and their responses to herbicides. Knowledge of their adapting behaviors will be critical to weed management strategies.

## Introduction

Climate components such as radiation, temperature and precipitation have a direct impact on the agriculture industry. Therefore, climate change could affect plant biophysiological processes and productivity^1^. An increase in the emission of greenhouse gasses (carbon dioxide-CO_2_, methane-CH_4_ and nitrous oxide-N_2_O_4_), aerosols, temperature and evaporation, as well as a decrease in precipitation will be important factors of future climate change^2^. These factors will influence other variables, such as different stresses (drought, salinity, etc), changes in pests’ life cycles and soils quality^3–6^.

The current atmospheric CO_2_ concentration, recorded at Mauna Loa Observatory, Hawaii, is 411 ppm^7^. Some studies have quantified a difference of 80 ppm in the CO_2_ concentration between urban and suburban areas^8,9^. According to emission scenarios on climate change as reported by the Intergovernmental Panel on Climate Change (IPCC), CO_2_ concentrations are predicted to be in the range between 600 to 1000 ppm at the end of the 21st century^10^.

Increased levels of CO_2_ in C_4_ weeds have less beneficial photosynthetic effects compared with C_3_ weeds because they already have a pathway for inhibiting photorespiration^11^. Different studies assessed that under current temperature conditions, elevated CO_2_ can increase aboveground biomass and productivity of some weeds through greater carbon availability and increases in photosynthesis^4,12,13^. However, increases in temperature, floods and drought can change the effect of elevated CO_2_ because plants respond to all environmental factors and one factor can influence the other factors^14^.

Some studies predicted a shift in the precipitation pattern and soil moisture deficiency^15–20^ For example, the amount of rainfall is expected to decrease in central Queensland, Australia, by 10–20% of the current rainfall by 2070^16^ and an average decrease of 2–5% is expected in all areas of Australia except the far north of Queensland by 2030^21^.

The change in the pattern of rainfall and temperature as a result of climate change can lead to the growth of C_4_ and thermophile weeds^4,22^. Some grass weeds, such as *Echinochloa* spp., *Setaria* spp., *Digitaria* spp., and *Sorghum halepense*, have expanded their distribution range because of climate change over past decades^23^. *E. colona* is highly sensitive to water stress^24,25^. Early stomata closing and reductions in CO_2_ assimilation and photosynthetic enzyme activities are the main responses of water deficit in plants^26^.

Plants have different pigments for absorbing light at different wavelengths, allowing a greater efficiency in light absorption in the photosynthetic process^27^. The number of photosynthetic pigments may change with environmental factors^28–31^. The effect of drought, CO_2_ concentration and temperature on different physiological processes in canola (*Brassica napus*) were studied and significant differences were reported between temperatures, CO_2_ concentrations and soil moistures for photosynthetic pigments content^31^.

Weeds are always among the problematic components in cropping systems. Therefore, understanding of weeds’ responses to climate change is essential for developing weed management strategies. Climate change can induce transformations and shifts in the weed flora and consequently changes their distribution and traits, making some of the opportunistic weeds invasive^32^. Response to climate change may vary, depending on the weed, region, latitude or soil^33^.

*Echinochloa colona* (L.) Link is a C_4_ annual summer grass native to Europe and India. It is a problematic weed in more than 60 countries and 35 crops^34^. In Australia, it has become problematic in summer fallows and crops such as maize (*Zea mays* L.), rice (*Oryza sativa* L.), cotton (*Gossypium hirsutum* L.), sugarcane (*Saccharum officinarum* L.) and sorghum (*Sorghum bicolor* L.)^35,36^. *E. colona* is an invasive weed with its vigorous growth traits and high seed production^37^. Each *E. colona* plant is capable of producing up to 42,000 seeds. Seeds can germinate at different ranges of soil temperature and moisture conditions^38^. The excessive use of glyphosate for *E. colona* control may exert an extreme selection pressure and lead to the evolution of resistant biotypes^39^. Glyphosate-resistant biotypes of *E. colona* have been reported in many cropping systems of Australia^35^.

Several studies showed that glyphosate efficacy is affected by soil moisture^40,41^. Although the effects of climate change and glyphosate efficacy are well documented for some weeds, little information is available on the response of resistant and susceptible *E. colona* biotypes to CO_2,_ soil moisture and glyphosate. In order to understand the impact of climate change on the resistant and susceptible *E. colona* biotypes and glyphosate efficacy in water deficient conditions, the current study was conducted. In our study, both glyphosate-resistant and susceptible biotypes have the same genetic background. The main objectives of the study were 1) to evaluate the growth and physiological responses of glyphosate-resistant and glyphosate-susceptible biotypes of *E. colona* to CO_2_ and soil moisture conditions, and 2) to evaluate the efficacy of glyphosate when applied on the plants of both biotypes (glyphosate-resistant and susceptible) growing in different soil moisture conditions.

## Materials and Methods

### Seed collection and development of glyphosate-resistant and susceptible biotypes

A suspected glyphosate-resistant biotype was collected from the research farm of the University of Queensland at Gatton, QLD, Australia (latitude 27.33°S, longitude 152.16°E and altitude 94 m.a.s.l.) in 2016. Resistance was confirmed in a screen-house study, in which plants were sprayed with different doses of glyphosate. Resistant and susceptible biotypes were developed through the cloning method as described by Mutti *et al*. (in press)^42^.

### Seedling preparation

Two experiments with two experimental runs/repeat (details given below) were conducted: one in growth chambers and the other in the screen-house. Seeds of both resistant and susceptible biotypes of *E. colona* were planted in plastic trays filled with a commercial potting mix and placed in the screen-house at the Gatton Campus of the University of Queensland, Australia, during the winter and autumn seasons of 2018. After one week, 2-leaf seedlings were transplanted into 15-cm-diameter plastic pots that were filled with a soil mix (potting mix and field soil at 1:1). Only one plant per pot was maintained. The pots were well watered and kept for 2 weeks in the screen-house with average minimum and maximum temperatures of 13.3–15.7 °C and 35.0–35.7 °C, respectively, for the two experimental runs. Two soil moisture conditions were applied after the three-leaf stage through the weighing method^43^. The 100% and 50% water holding capacity (WHC) were considered as well-watered and water-stressed conditions, respectively.

### Experiment I. CO_2_ and soil moisture: A growth chamber study

The resistant and susceptible biotypes of *E. colona* were grown in pots placed in two growth chambers set at ambient CO_2_ (450 ppm) and elevated CO_2_ (750 ppm) under well-watered and water-stressed conditions. The temperature in both growth chambers was set at 30/20 °C (12 h light/12 h dark), optimum conditions for *E. colona*^42^. This experiment was conducted in a completely randomized design with six replications. Physiological and growth characteristics such as plant height, dry biomass, number of leaves, tillers and inflorescences per plant were measured at an interval of 10 days. Number of seeds per plant and photosynthetic pigments were measured at the end of the experiment.

Photosynthetic pigment content was measured with a portable meter and an extractable chlorophyll method^44^. Two middle leaves of each plant were marked and the relative chlorophyll content was measured using a SPAD meter. At the end of the experiment, the same leaves were used for measuring the amount of chlorophyll *a*, *b* and carotenoids using the extractable method proposed by Hiscox and Israelstam^45^. For each sample, around 0.05 g of fresh leaves were weighed and after adding 5 ml dimethylsulfoxide (DMSO), all samples were placed in a water bath at 65 °C for 45 minutes^45^. The final solution of samples was measured with a spectrophotometer (Shimadzu, visible spectrophotometer, UV-2550) for detecting the amount of pigments using the wavelengths of 470, 645 and 663 nm for carotenoids, chlorophyll *b* and chlorophyll *a*, respectively^46^. Chlorophyll content was estimated by measuring the correlation of the extractable method and the portable meter value^47^.

### Experiment II. Glyphosate efficacy and soil moisture: A screen-house study

Plants of both biotypes were grown in the screen-house at two soil moisture conditions: well-watered (irrigated daily) and water-stressed (irrigation stopped 2 weeks before glyphosate application). Plants were treated with different glyphosate doses (0, 180, 360, 720 and 1440 g a.e. ha^−1^) at the 3–4 leaf stage with a Research Track Sprayer (using 108 L water solution/ha). Flat fan nozzles were used in the sprayer. After 24 hours of spraying, all plants were well watered daily. At 2 weeks after spraying, plant survival data were taken with the criterion of survival being at least one green leaf. Surviving plants were cut from the soil surface, placed in paper bags and dried in an oven at 70 °C for 48 h for measuring dry biomass. The experiment was conducted in a randomized complete block design with eight replications.

### Statistical analyses

Both experiments were conducted twice (experimental runs). In the experiments, whenever no significant interaction was observed between experimental runs and treatments, data from both runs were pooled for analysis of variance (ANOVA). Results were reported separately when the interaction of experimental run × treatment was significant. SAS (version 9.0.3) was used for ANOVA. Data from both experiments met the assumptions of homogeneity of variance and normality, and did not need transformation.

In experiment I, a three-parameter sigmoidal model was fitted to the height data:

1 $$f=a/(1+\exp (-(x-{x}_{50})/b)$$

In this equation, *f* represents height at time *x*, *a* is the maximum height at a given time, *x*_50_ is the time (days) required to attain 50% height of the maximum height and *b* indicates the slope.

Leaf, tiller, and inflorescence numbers per plant were modeled using a two-parameter exponential growth equation:

2 $$f=a\times \exp (b\times x)$$

In this equation, *f* represents the number of leaves, tillers or inflorescences at time *x*, *a* is the intercept and *b* indicates the slope.

## Results

### Experiment I. CO_2_ and soil moisture: A growth chamber study

#### Plant height

**Figure 1:**
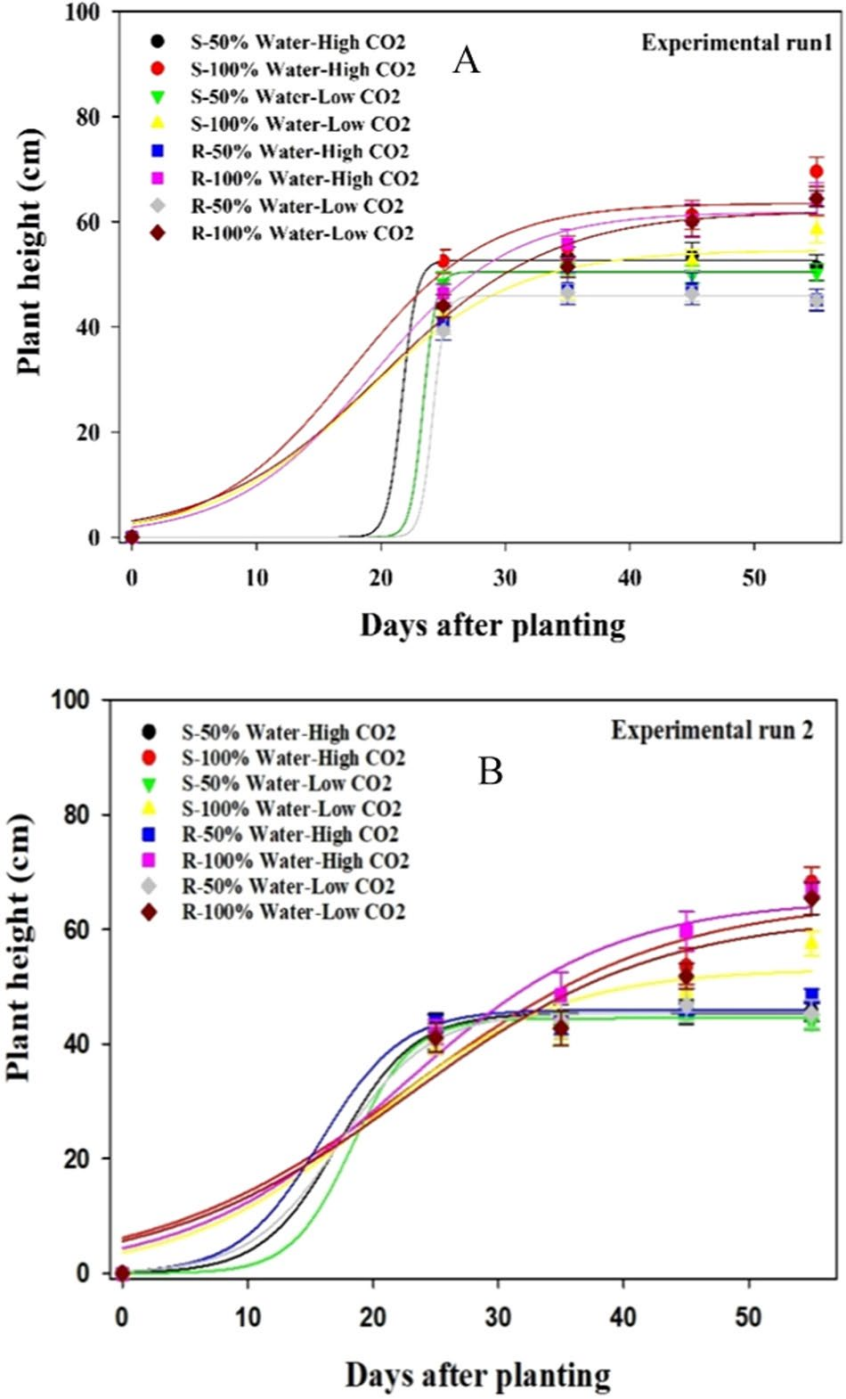
Effect of different CO_2_ concentrations and soil moisture on the height of susceptible (S) and resistant (R) biotypes of *Echinochloa colona*. (**A**) first experimental run (**B**) second experimental run. High CO_2_ and Low CO_2_ denote 750 ppm and 450 ppm CO_2_ concentration, respectively. 50% water represents water-stress treatment and 100% water represents well-water treatment. Modeled with the use of equation *f* = *a*/(1 + exp (−(*x* − *x*_50_)/*b*). In this equation *f* represents height at time *x*, *a* is the maximum height at a given time, *x*_50_ is the time (days) required for 50% height and *b* indicates the slope. Estimated parameters are given in Table 1. Vertical bars represent the standard error of means (*Experiment I*).

Soil moisture and elevated CO_2_ affected the plant height of both resistant and susceptible biotypes. Plants grown in the well-watered treatment were taller than those grown in the water-stressed treatment at both CO_2_ concentrations (Fig. 1a,b). In the well-watered treatment, 55 days after planting, the height of the susceptible biotype at elevated CO_2_ was increased by ~16% (means of experimental runs) compared to plants grown at ambient CO_2_, but there was no significant increase in the resistant biotype. In the water-stressed treatment, no significant difference was observed between the height of the resistant and susceptible biotypes at both CO_2_ concentrations. Compared with the well-watered treatment, the height of both resistant and susceptible plants was decreased by ~29% in the water-stressed treatment at elevated CO_2_ (Fig. 1a,b). The maximum height was observed for the susceptible biotype in the well-watered treatment at elevated CO_2_.

The comparison of the slope (*b* parameter) of the curves shows that in the water-stressed treatment, plant height was mostly constant from 25 days after planting to weed maturity and this was true at both CO_2_ concentrations (Fig. 1a,b; Table 1).

**Table 1:** Effect of different CO_2_ concentrations and soil moisture on the height of the glyphosate-resistant (R) and glyphosate-susceptible (S) biotypes of *Echinochloa colona* (*Experiment I*).

Treatments	Parameters			
	a	b	X_0_	R^2^
**Experimental run 1**				
S- 50% Water- High CO_2_	52.6 ± 0.8	0.5 ± 0.0	21.6 ± 8.4	0.99
S- 100% Water- High CO_2_	63.5 ± 4.4	5.4 ± 3.3	17.5 ± 5.0	0.94
S- 50% Water- Low CO_2_	50.4 ± 0.3	0.5 ± 0.2	23.3 ± 4.8	0.99
S- 100% Water- Low CO_2_	54.6 ± 3.6	6.1 ± 2.9	18.6 ± 3.7	0.97
R- 50% Water- High CO_2_	Model could not fit			
R- 100% Water- High CO_2_	61.8 ± 2.2	5.5 ± 1.8	19.3 ± 2.1	0.99
R- 50% Water- Low CO_2_	45.9 ± 0.6	0.4 ± 0.0	24.1 ± 0.0	0.99
R- 100% Water- Low CO_2_	62.0 ± 3.8	6.8 ± 2.6	20.1 ± 2.7	0.98
**Experimental run 2**				
S- 50% Water- High CO_2_	45.4 ± 0.2	3.0 ± 0.9	17.2 ± 2.4	0.99
S- 100% Water- High CO_2_	65.2 ± 3.5	10.2 ± 6.7	23.0 ± 7.2	0.92
S- 50% Water- Low CO_2_	44.1 ± 0.4	2.4 ± 6.0	18.3 ± 6.4	0.99
S- 100% Water- Low CO_2_	53.1 ± 5.7	7.5 ± 4.2	19.7 ± 4.8	0.95
R- 50% Water- High CO_2_	45.9 ± 1.4	3.2 ± 2.9	15.7 ± 8.4	0.99
R- 100% Water- High CO_2_	65.2 ± 6.5	8.4 ± 3.6	23.2 ± 3.5	0.99
R- 50% Water- Low CO_2_	45.5 ± 0.7	3.6 ± 1.7	17.3 ± 3.6	0.99
R- 100% Water- Low CO_2_	62.4 ± 11.5	9.9 ± 6.1	22.9 ± 6.4	0.93

High CO_2_ and Low CO_2_ denote 750 ppm and 450 ppm CO_2_ concentration, respectively. 50% water represents water-stress treatment and 100% water represents well-water treatment.

#### Number of leaves per plant

**Figure 2:**
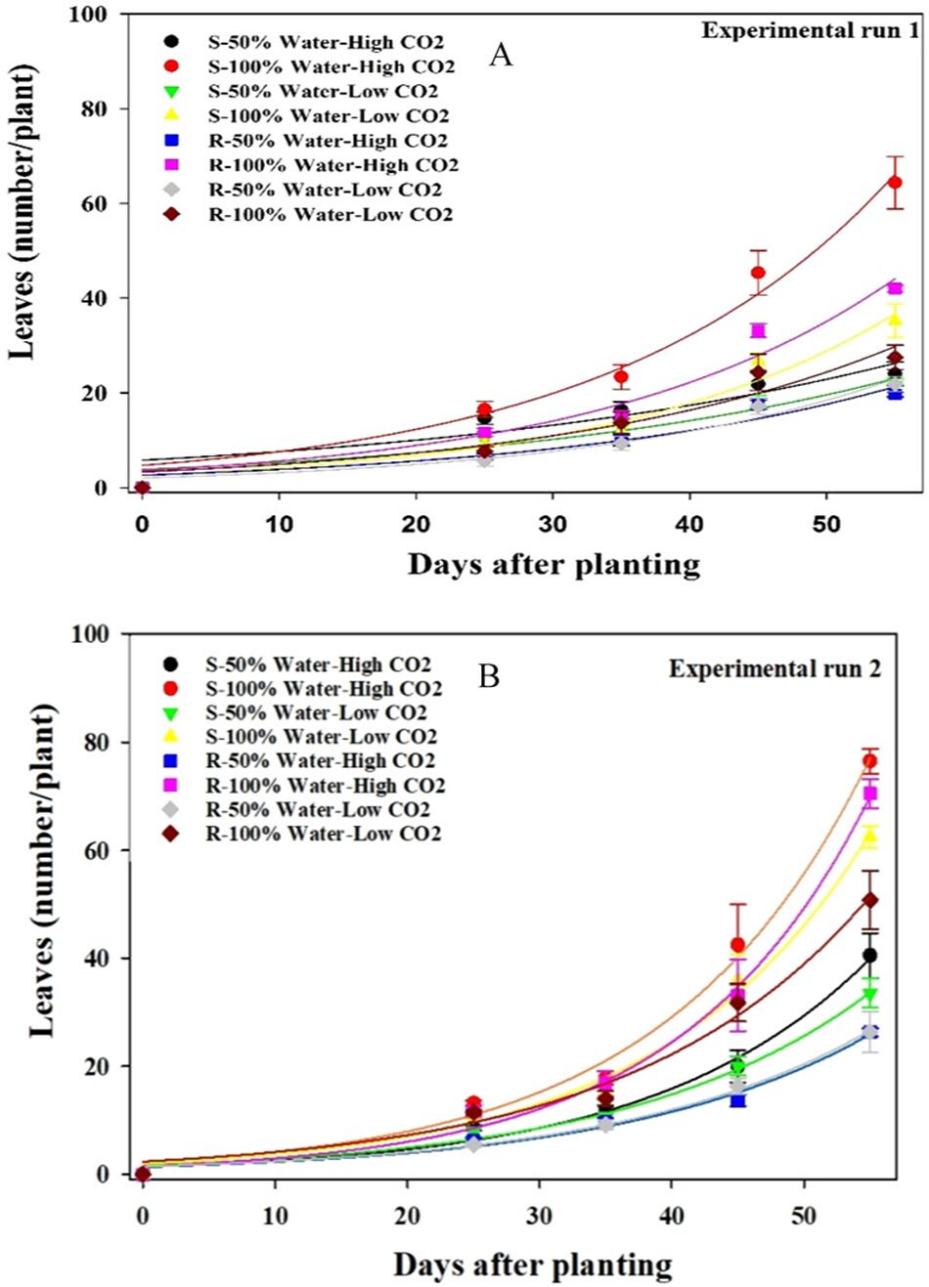
Effect of different CO_2_ concentrations and soil moisture on the number of leaves per plant in susceptible (S) and resistant (R) biotypes of *Echinochloa colona* (**A**) first experimental run (**B**) second experimental run. High CO_2_ and Low CO_2_ denote 750 ppm and 450 ppm CO_2_ concentration, respectively. 50% water represents water-stress treatment and 100% water represents well-water treatment. Modeled with the use of equation *f* = *a* * exp(b * x). In this equation *f* represents the number of leaves per plant at time *x*, *a* is a constant amount and *b* indicates the slope. Estimated parameters are given in Table 2. Vertical bars represent the standard error of means (*Experiment I*).

At elevated CO_2_, the number of leaves per plant in the well-watered treatment was significantly higher than in the water-stressed treatment at 55 days after planting; 55% and 58% greater for susceptible and resistant biotypes, respectively (Fig. 2a,b). In the water-stressed condition, the susceptible biotype at ambient and elevated CO_2_ produced 7% and 28% greater number of leaves, respectively, than the resistant biotype (Fig. 2a,b). The comparison of the slope (*b* parameter) shows that the increase in the number of leaves in the well-watered treatment was faster than in the water-stressed treatment (Table 2).

**Table 2:** Effect of different CO_2_ concentrations and soil moisture on the number of leaves per plant in the glyphosate-resistant (R) and glyphosate-susceptible (S) biotypes of *Echinochloa colona* (*Experiment I*).

Treatments	Parameters		
	a	b	R^2^
**Experiment 1**			
S- 50% Water- High CO_2_	5.70 ± 2.40	0.02 ± 0.009	0.84
S- 100% Water- High CO_2_	4.70 ± 1.30	0.04 ± 0.005	0.98
S- 50% Water- Low CO_2_	3.82 ± 1.30	0.03 ± 0.007	0.92
S- 100% Water- Low CO_2_	2.57 ± 1.05	0.04 ± 0.008	0.96
R- 50% Water- High CO_2_	2.62 ± 1.11	0.03 ± 0.008	0.92
R- 100% Water- High CO_2_	3.55 ± 1.49	0.04 ± 0.008	0.95
R- 50% Water- Low CO_2_	2.03 ± 0.73	0.04 ± 0.007	0.96
R- 100% Water- Low CO_2_	3.29 ± 1.40	0.04 ± 0.009	0.92
**Experiment 2**			
S- 50% Water- High CO_2_	1.35 ± 0.39	0.06 ± 0.005	0.98
S- 100% Water- High CO_2_	2.17 ± 0.54	0.06 ± 0.004	0.99
S- 50% Water- Low CO_2_	1.65 ± 0.36	0.05 ± 0.004	0.99
S- 100% Water- Low CO_2_	1.98 ± 0.42	0.06 ± 0.004	0.99
R- 50% Water- High CO_2_	1.35 ± 0.39	0.05 ± 0.005	0.98
R- 100% Water- High CO_2_	1.48 ± 0.34	0.07 ± 0.004	0.99
R- 50% Water- Low CO_2_	1.43 ± 0.26	0.05 ± 0.003	0.99
R- 100% Water- Low CO_2_	2.37 ± 0.71	0.05 ± 0.005	0.98

High CO_2_ and Low CO_2_ denote 750 ppm and 450 ppm CO_2_ concentration, respectively. 50% water represents water-stress treatment and 100% water represents well-water treatment.

#### Number of tillers per plant

**Figure 3:**
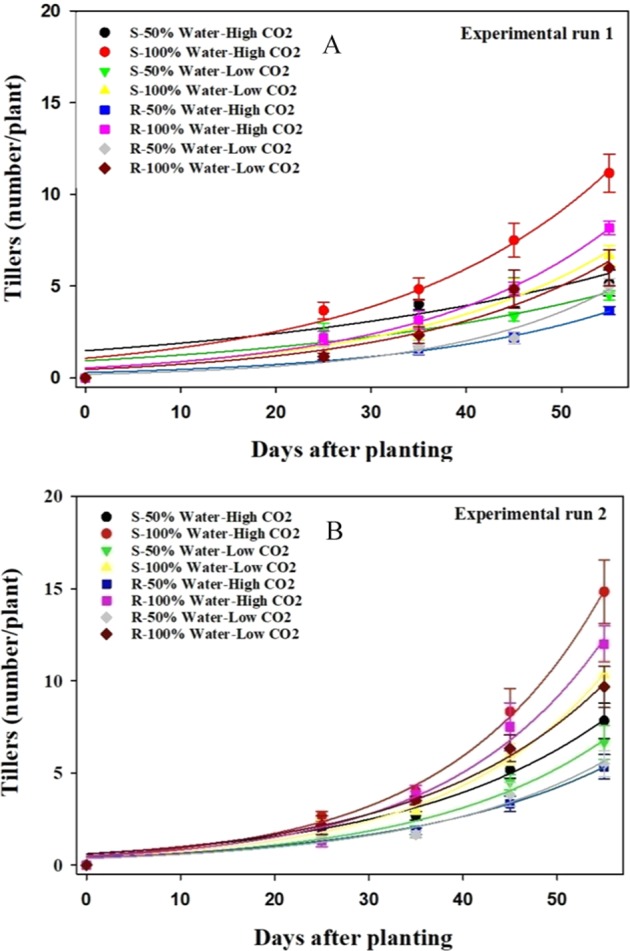
Effect of different CO_2_ concentrations and soil moisture on the number of tillers per plant of susceptible (S) and resistant (R) biotype of *Echinohcloa colona*. (**A**) first experimental run (**B**) second experimental run. High CO_2_ and Low CO_2_ denote 750 ppm and 450 ppm CO_2_ concentration, respectively. 50% water represents water-stress treatment and 100% water represents well-water treatment. Modeled with the use of equation *f* = *a* * exp*(b* * *x*). In this equation *f* represents the number of tillers per plant at time *x*, *a* is a constant amount and *b* indicates the slope. Estimated parameters are given in Table 3. Vertical bars represent the standard error of means (*Experiment I*).

Regardless of moisture condition, elevated CO_2_ increased the number of tillers in both biotypes; however, this increase was more obvious in the well-watered treatment than in the water-stressed treatment (Fig. 3a,b, Table 3). At elevated CO_2_, the susceptible biotype produced 23% more tillers than the resistant biotype in the well-watered treatment (Fig. 3a,b).

**Table 3:** Effect of different CO_2_ concentrations and soil moisture on the number of tillers per plant in the glyphosate-resistant (R) and glyphosate-susceptible (S) biotypes of *Echinochloa colona* (*Experiment I*).

Treatments	Parameters		
	a	b	R^2^
**Experiment 1**			
S- 50% Water- High CO_2_	1.48 ± 0.69	0.020 ± 0.010	0.78
S- 100% Water- High CO_2_	1.07 ± 0.27	0.040 ± 0.005	0.97
S- 50% Water- Low CO_2_	0.93 ± 0.38	0.029 ± 0.008	0.87
S- 100% Water- Low CO_2_	0.55 ± 0.18	0.045 ± 0.006	0.96
R- 50% Water- High CO_2_	0.29 ± 0.08	0.045 ± 0.005	0.97
R- 100% Water- High CO_2_	0.54 ± 0.12	0.049 ± 0.004	0.98
R- 50% Water- Low CO_2_	0.20 ± 0.10	0.057 ± 0.010	0.95
R- 100% Water- Low CO_2_	0.46 ± 0.21	0.047 ± 0.009	0.94
**Experiment 2**			
S- 50% Water- High CO_2_	0.62 ± 0.21	0.046 ± 0.007	0.96
S- 100% Water- High CO_2_	0.50 ± 0.09	0.061 ± 0.003	0.99
S- 50% Water- Low CO_2_	0.38 ± 0.15	0.052 ± 0.008	0.96
S- 100% Water- Low CO_2_	0.41 ± 0.07	0.058 ± 0.003	0.99
R- 50% Water- High CO_2_	0.41 ± 0.14	0.046 ± 0.007	0.96
R- 100% Water- High CO_2_	0.46 ± 0.15	0.059 ± 0.006	0.98
R- 50% Water- Low CO_2_	0.35 ± 0.13	0.050 ± 0.007	0.97
R- 100% Water- Low CO_2_	0.60 ± 0.13	0.050 ± 0.004	0.99

High CO_2_ and Low CO_2_ denote 750 ppm and 450 ppm CO_2_ concentration, respectively. 50% water represents water-stress treatment and 100% water represents well-water treatment.

#### Number of inflorescences per plant

**Figure 4:**
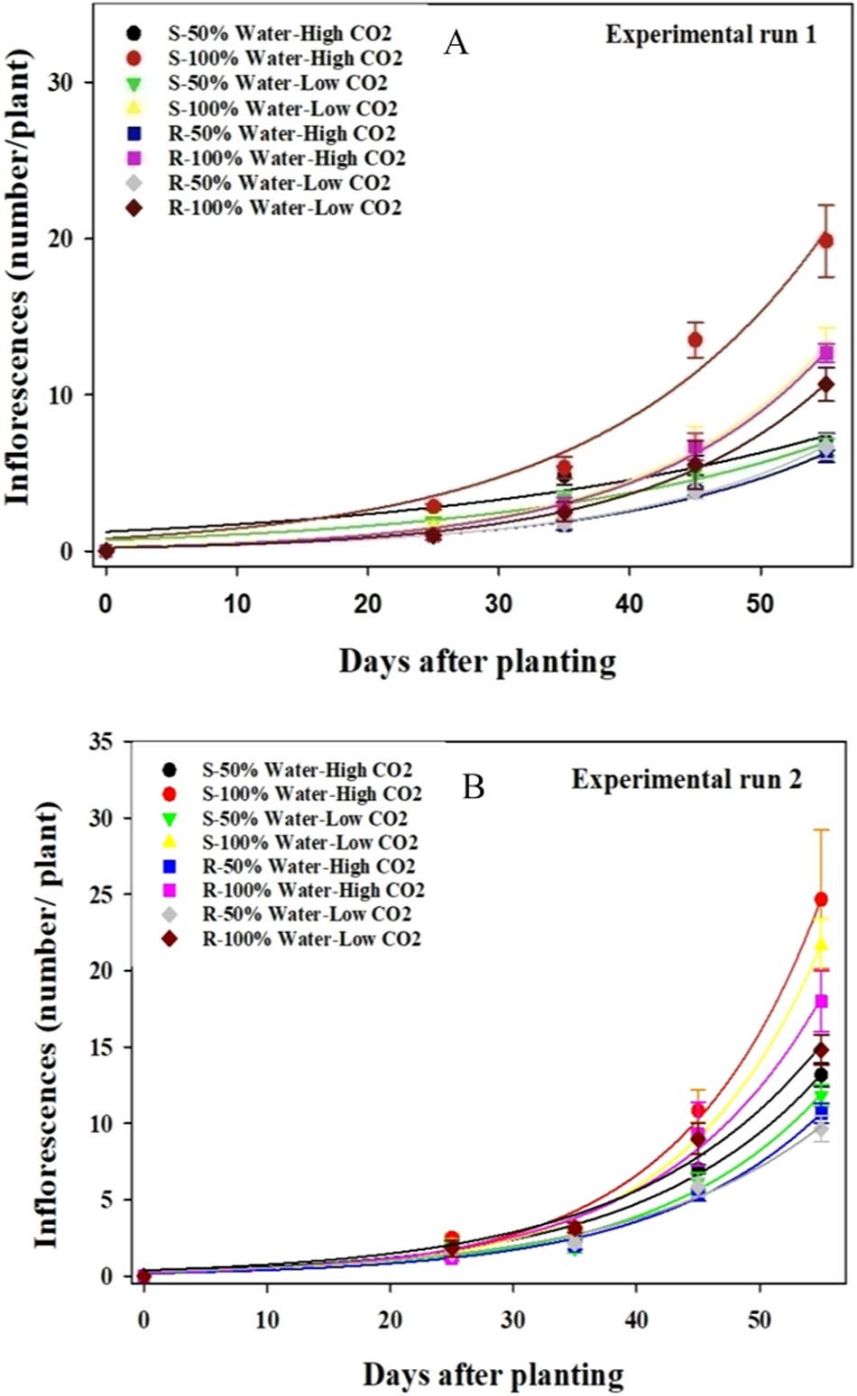
Effect of different CO_2_ concentrations and soil moisture on the number of inflorescences per plant of susceptible (S) and resistant (R) biotype of *Echinochloa colona*. (**A**) First experimental run (**B**) second experimental run. High and Low CO_2_ denote 750 and 450 ppm CO_2_ concentration, respectively. 50% water represents water-stress treatment and 100% water represents well-water treatment. Modelled with the use of equation *f* = *a* * exp (*b* * *x*). In this equation f represents the number of inflorescences per plant at time *x, a* is a constant amount and *b* indicates the slope. Estimated parameters are given in Table 4. Vertical bars represent the standard error of means (*Experiment I*).

In both biotypes, the increase in soil moisture and CO_2_ resulted in a significant increase in the number of inflorescences per plant; however, the comparison of the slope (*b* parameter) of the curves shows that water availability had a more pronounced effect on the number of inflorescences per plant (Table 4). The susceptible biotype produced more inflorescence numbers than the resistant biotype at both CO_2_ concentrations in the well-watered condition (Fig. 4a,b). At both CO_2_ concentrations, the lowest number of inflorescences was observed in the resistant biotype under water-stressed conditions (Fig. 4a,b).

**Table 4:** Effect of different CO_2_ concentrations and soil moisture on the number of inflorescences per plant in the glyphosate-resistant (R) and glyphosate-susceptible (S) biotypes of *Echinochloa colona* (*Experiment I*).

Treatments	Parameters		
	a	b	R^2^
**Experiment 1**			
S- 50% Water- High CO_2_	0.24 ± 0.49	0.032 ± 0.008	0.90
S- 100% Water- High CO_2_	0.81 ± 0.35	0.058 ± 0.008	0.97
S- 50% Water- Low CO_2_	0.70 ± 0.21	0.041 ± 0.006	0.96
S- 100% Water- Low CO_2_	0.25 ± 0.08	0.071 ± 0.006	0.99
R- 50% Water- High CO_2_	0.23 ± 0.06	0.059 ± 0.005	0.98
R- 100% Water- High CO_2_	0.24 ± 0.05	0.070 ± 0.004	0.99
R- 50% Water- Low CO_2_	0.21 ± 0.04	0.062 ± 0.003	0.99
R- 100% Water- Low CO_2_	0.20 ± 0.03	0.072 ± 0.003	0.99
**Experiment 2**			
S- 50% Water- High CO_2_	0.31 ± 0.10	0.068 ± 0.006	0.98
S- 100% Water- High CO_2_	0.19 ± 0.06	0.087 ± 0.006	0.99
S- 50% Water- Low CO_2_	0.20 ± 0.08	0.074 ± 0.008	0.98
S- 100% Water- Low CO_2_	0.17 ± 0.04	0.087 ± 0.005	0.99
R- 50% Water- High CO_2_	0.19 ± 0.05	0.073 ± 0.005	0.99
R- 100% Water- High CO_2_	0.24 ± 0.08	0.078 ± 0.006	0.99
R- 50% Water- Low CO_2_	0.29 ± 0.10	0.064 ± 0.006	0.98
R- 100% Water- Low CO_2_	0.39 ± 0.16	0.066 ± 0.007	0.98

High CO_2_ and Low CO_2_ denote 750 ppm and 450 ppm CO_2_ concentration, respectively. 50% water represents water-stress treatment and 100% water represents well-water treatment.

#### Number of seeds per plant

In the well-watered treatment, the susceptible biotype produced more seeds than the resistant one under both CO_2_ concentrations (Table 5). However, no significant difference was observed between their seed production in the water-stressed condition. The decrease in water availability (by 50%) led to a decrease in seed production in the resistant and susceptible biotypes by 67% and 88% at 450 ppm and 45% and 72% at 750 ppm CO_2_, respectively. Increasing the CO_2_ concentration did not significantly change the number of seeds per plant in both biotypes.

**Table 5:** Effect of different CO_2_ concentrations and soil moisture conditions on the number of seeds per plant in the glyphosate-resistant and glyphosate-susceptible biotypes of *Echinochloa colona* (*Experiment I*).

CO_2_ concentration	Seed production (number per plant)			
	Well water		Water stress	
	Susceptible	Resistant	Susceptible	Resistant
450 ppm	2561	1773	542	585
750 ppm	2410	1398	666	601
	LSD (0.05%) = 433			

#### Total dry shoot biomass

An increase in water availability and CO_2_ concentration resulted in an increase in shoot biomass of both biotypes but the effect of water availability was more than CO_2_ concentration (Table 6). The highest amount of shoot biomass was observed for the susceptible biotype in the well-watered treatment under elevated CO_2_ and the lowest biomass was observed in the water-stressed treatment under ambient CO_2_ concentration in the susceptible biotype. In the well-watered condition, the biomass of the resistant and susceptible biotypes increased by 12% and 47%, respectively, at elevated CO_2_ compared with the ambient CO_2_ concentration. Water stress reduced the biomass of the resistant and susceptible biotypes by 73% and 77%, respectively, at elevated CO_2_. Under ambient CO_2_, water stress decreased the total dry biomass of the resistant and susceptible biotypes by 70% and 64%, respectively. The response of the susceptible biotypes was more evident compared with the resistant biotypes in both soil moisture levels and CO_2_ concentrations.

**Table 6:** Effect of carbon dioxide (CO_2_) concentrations and soil moisture on dry biomass of the glyphosate-resistant and glyphosate-susceptible biotypes of *Echinochloa colona* (*Experiment I*).

CO_2_ concentration	Dry biomass (g plant^−1^)			
	Well water		Water stress	
	Susceptible	Resistant	Susceptible	Resistant
**Experimental run 1**				
450 ppm	1.09	1.56	0.47	0.51
750 ppm	2.71	1.93	0.61	0.52
	LSD (0.05%) = 0.48			
**Experimental run 2**				
450 ppm	2.93	3.58	0.81	0.95
750 ppm	4.45	3.77	1.07	1.01
	LSD (0.05%) = 0.67			

#### Photosynthetic pigments

In both experimental runs, significant differences were found between soil moisture treatments for the content of photosynthetic pigments, while no significant differences were observed between CO_2_ concentrations (Table 7). The well-watered condition significantly increased the amount of total chlorophyll by 23% and 25% in the resistant and susceptible biotypes, respectively, in the ambient CO_2_ condition.

**Table 7:** Content of photosynthetic pigment of the glyphosate-resistant and glyphosate-susceptible biotypes of *Echinochloa colona* grown at different carbon dioxide (CO_2_) concentrations and soil moisture in growth chambers (*Experiment I*).

Photosynthetic pigments content (mg g^−1^ dry weight)		Well water		Water stress	
		Susceptible	Resistant	Susceptible	Resistant
**Experimental run 1**					
Carotenoids	450 ppm	1.77	1.77	1.44	1.43
	750 ppm	1.81	1.79	1.47	1.44
	LSD (0.05%) = 0.07				
Chlorophyll *a*	450 ppm	30.55	30.53	24.94	24.69
	750 ppm	31.34	30.88	25.36	24.86
	LSD (0.01%) = 1.30				
Chlorophyll *b*	450 ppm	6.76	6.76	5.21	5.14
	750 ppm	6.96	6.86	5.33	5.19
	LSD (0.05%) = 0.36				
Chlorophyll total	450 ppm	37.32	37.3	30.16	29.84
	750 ppm	38.2	37.75	30.7	30.06
	LSD (0.05%) = 1.66				
**Experimental run 2**					
Carotenoids	450 ppm	2.42	2.39	1.71	1.8
	750 ppm	2.41	2.46	1.71	1.8
	LSD (0.05%) = 0.19				
Chlorophyll *a*	450 ppm	41.87	41.35	29.49	31.09
	750 ppm	41.69	42.54	29.6	31.03
	LSD (0.01%) = 3.32				
Chlorophyll *b*	450 ppm	9.9	9.76	6.47	6.9
	750 ppm	9.85	10.09	6.5	6.9
	LSD (0.05%) = 0.92				
Chlorophyll total	450 ppm	51.78	51.11	35.97	38.01
	750 ppm	51.55	52.63	36.11	37.93
	LSD (0.05%) = 4.25				

### Experiment II. Glyphosate efficacy and soil moisture: A screen-house study

Biomass data showed that glyphosate efficacy was significantly decreased in the water-stressed condition at all glyphosate doses (Table 8). The resistant biotype in the well-watered treatment had 19% survival at 1440 g ha^−1^ glyphosate (twice of the recommended dose), but this survival degree increased in the water-stressed treatment by 62% (Table 8). For the susceptible biotype, plant biomass decreased by 62% and 92% at 720 and 1440 g ha^−1^ glyphosate, respectively, in the water-stressed condition, while at the same herbicide doses, no plant survived in the well-water condition (Table 9).

**Table 8:** The effect of different doses of glyphosate and soil moisture on plant survival of the glyphosate-resistant and glyphosate-susceptible biotypes of *Echinochloa colona* (*Experiment II*).

Dose (g a.e. ha^−1^)	Dry biomass (g plant^−1^)			
	Well-water		Water-stress	
	Susceptible	Resistant	Susceptible	Resistant
control	100.0	100.0	100.0	100.0
180	93.8	100.0	93.8	100.0
360	93.8	93.8	93.8	100.0
720	0	75.0	87.5	100.0
1440	0	18.8	25.0	81.2
	LSD (0.05) = 16.43			

Well-water represents daily irrigation, in water-stress irrigation stopped 2 weeks before glyphosate application.

**Table 9:** The effect of different doses of glyphosate and soil moisture on total biomass of the glyphosate-resistant and glyphosate-susceptible biotypes of *Echinochloa colona* (*Experiment II*).

Dose (g a.e. ha^−1^)	Dry biomass (g plant^−1^)			
	Well-water		Water-stress	
	Susceptible	Resistant	Susceptible	Resistant
0	0.47	0.40	0.32	0.30
180	0.24 (48%)	0.32 (24%)	0.16 (50%)	0.29 (5%)
360	0.17 (63%)	0.19 (54%)	0.13 (58%)	0.20 (35%)
720	0 (100%)	0.11 (71%)	0.12 (62%)	0.14 (52%)
1440	0 (100%)	0.030 (93%)	0.02 (92%)	0.06 (80%)
	LSD = 0.09			

The reduction (%) was presented in parenthesis.

Well-water represents daily irrigation, in water-stress irrigation stopped 2 weeks before glyphosate application.

## Discussion

Elevated CO_2_ resulted in taller plants of both susceptible and resistant *E. colona* biotypes with more tillers, leaves, and biomass, but seed production was not affected by the increased CO_2_ concentration. Generally, elevated CO_2_, when considered alone, leads to increased numbers of leaves and inflorescences, height and total biomass, which could be attributed to increased photosynthesis and water use efficiency and decreased transpiration through reducing stomatal conductance^48–52^. While some studies reported an increase in seed production by elevated CO_2_ ^4,5^, our study found no significant difference. In C_4_ species, because of their ability to concentrate CO_2_ via their photosynthesis pathway^53^, increasing the external CO_2_ concentration has little effect on net photosynthesis^54^, but it should not be assumed that C_4_ plants do not have the ability to use high CO_2_ amounts^55^.

Water deficit is one of the most concerning issues surrounding climate change and may interfere with plant growth and development. The current study observed that water deficit resulted in the reduction of growth parameters and consequently seed production, especially for the susceptible biotype. Other studies also considered the importance of water deficiency on weed growth^56,57^. The amount of photosynthetic pigments was significantly decreased by the reduction in water availability. Water stress can affect the synthesis of chlorophyll, the electron transport chain and consequently, synthesis of all proteins and enzymes, such as carboxylase, that have essential roles in photosynthesis^29,58^. How the pigment amount is affected may be related to the competitive ability of weeds, as a species with higher amounts of photosynthetic pigments may be more competitive^46^.

The interaction effect of soil moisture and CO_2_ concentration significantly influenced all measured growth parameters and seed production. The effect of elevated CO_2_ in increasing plant growth is likely to happen at the optimum temperature for growth and sufficient water availability^14,59^. In the current study, the effect of soil moisture and CO_2_ concentration was examined at the optimum temperature for *E. colona*. Water availability was found to affect weed growth more than CO_2_ concentration. Elevated CO_2_ can be helpful for the vegetative growth of plants but cannot compensate for the adverse effect of water stress on them^56^. Leakey *et al*. suggested that the increase in the growth potential of C_4_ plants by elevated CO_2_ depends on the decrease in water use and reduction in drought stress, and not by the direct effect of increased photosynthesis^57^. The water requirement of weeds will increase under rising CO_2_ and temperature^56^. Plants in water stress conditions cannot properly use high CO_2_ concentration as much as those that are well watered, due to the lower stomatal conductance caused by less guard cell turgescence. Therefore, CO_2_ uptake will decrease in these plants^12,60^. The difference in seed production between the resistant and susceptible biotypes was not significant in the water-stressed condition at both CO_2_ concentrations. In the water-stressed condition, increasing CO_2_ concentration via decreasing stomatal conductance and increasing water use efficiency may allow plants to produce more seeds, but total biomass may always be lower compared with plants grown in well-watered conditions^60^.

In both biotypes, growth and seed production were enhanced by increasing CO_2_ concentration and water availability. In the well-watered treatment, the stimulation of photosynthesis from increased CO_2_ concentration in our study was more evident in the susceptible than in the resistant biotype. Despite higher vegetative growth of the susceptible biotype, no difference was observed in seed production between biotypes in the water-stressed treatment. It can be concluded that the resistant biotype allocated more photosynthetic resources to seed production compared with vegetative growth in the stressed condition. Potvin (1986) mentioned a strategy of investing more resources to inflorescence development (versus leaves) in *E. crus-galli* plants due to the importance and critical role of seed production in population dynamics^61^. The link between plant size and evolutionary fitness is the ability of plants to allocate resources to reproduction^3^.

In Experiment II, reducing soil moisture content resulted in a decrease in the efficacy of glyphosate. This response could be caused by less absorption and translocation of glyphosate as the herbicide is mainly translocated by vascular transportation^62^. Tanpipat *et al*. also claimed that water stress via reducing leaf area can affect glyphosate uptake^41^. The requirement of high doses of glyphosate in the water-stressed condition may be related to the increase in the concentration gradient across the cuticle, consequently leading to more glyphosate uptake^63^. Using high glyphosate rates in water stress conditions may cause a high risk of producing resistant biotypes.

It is predicted that climate change will have a significant impact on weed management strategies in the future^22^. The latest studies on climate change in regards to weeds suggest that focusing on drought-resistant weed biotypes seems to be a more logical resolution than other biotypes. Understanding weed fitness could help to predict the dynamics of herbicide-resistant weeds and their management^64^. Species that showed adaptation to drought conditions were less adversely affected by climate change and were able to compete better in dry soil rather than species which adapted to wet soil moisture conditions^65^. In addition, the current study observed that herbicide efficacy was reduced by decreasing water availability. Therefore, more studies on herbicide efficacy in climate change conditions should be considered.

## Conclusions

Environmental changes can affect the physiological and growth processes of weeds and their responses to herbicides. *E. colona* biotypes used in this study showed greater vegetative growth in response to elevated CO_2_. In both biotypes, seed production and photosynthesis pigments were not affected by the increased CO_2_ concentration. However, the water-stress condition caused a significant decrease in growth parameters, seed production and glyphosate efficacy in both biotypes. The results of this study suggest that the predicted climate change can make this weed more noxious and competitiveness. It is possible that increased vegetative growth of weeds combined with water deficiency caused by climate change reduces the herbicide uptake and translocation and consequently, decrease herbicide efficacy. More studies based on different climate change factors need to be conducted to elucidate the role of environmental parameters and nutrition on opportunistic weeds’ responses. A better understanding of how weeds respond to climate change based on known tolerance ranges and climatic selection pressures is suggested for developing effective weed management strategies.

## References

[1] Nelson GC (2014). Climate change effects on agriculture: Economic responses to biophysical shocks. Proc. Natl. Acad. Sci..

[2] Chauhan, B. S., Mahajan, G., Randhawa, R. K., Singh, H. & Kang, M. S. *Global warming and its possible impact on agriculture in India*. In *Advances in agronomy*. Vol. 123 65-121 (Elsevier, 2014).

[3] Clements DR, Ditommaso A (2011). Climate change and weed adaptation: can evolution of invasive plants lead to greater range expansion than forecasted?. Weed Res..

[4] Peters K, Breitsameter L, Gerowitt B (2014). Impact of climate change on weeds in agriculture: a review. Agron. Sustain. Dev..

[5] Thomson LJ, Macfadyen S, Hoffmann AA (2010). Predicting the effects of climate change on natural enemies of agricultural pests. Biol. Control..

[6] Ripple, W. J., Wolf, C., Newsome, T. M., Barnard, P. & Moomaw, W. R. World scientists’ warning of a climate emergency. *BioScience*, 10.1093/biosci/biz088 (2019).

[7] - National Oceanic Atmospheric Administration Research. Recent Monthly Average Mauna Loa CO_2_ Report. Available at, www.esrl.noaa.gov/gmd/ccgg/trends/ (NOAA, 2019)

[8] Idso CD, Idso SB, Balling RC (2001). An intensive two-week study of an urban CO_2_ dome in Phoenix, Arizona, USA. Atmos. Environ..

[9] Ziska LH, Faulkner S, Lydon J (2004). Changes in biomass and root: shoot ratio of field-grown Canada thistle (*Cirsium arvense*), a noxious, invasive weed, with elevated CO_2_: implications for control with glyphosate. Weed Sci..

[10] Pachauri, R. K. *et al*. *Climate change 2014: synthesis report. Contribution of Working Groups I, II and III to the fifth assessment report of the Intergovernmental Panel on Climate Change*. (IPCC, 2014).

[11] Ghannoum O, von Caemmerer S, Barlow EW, Conroy JP (1997). The effect of CO_2_ enrichment and irradiance on the growth, morphology and gas exchange of a C_3_ (*Panicum laxum*) and a C_4_ (*Panicum antidotale*) grass. Funct. Plant Biol..

[12] Hampton JG, Conner AJ, Boelt B, Chastain TG, Rolston P (2016). Climate Change: Seed Production and Options for Adaptation. Agricult..

[13] Wand SJ, Midgley GF, Jones MH, Curtis PS (1999). Responses of wild C_4_ and C_3_ grass (Poaceae) species to elevated atmospheric CO_2_ concentration: a meta‐analytic test of current theories and perceptions. Glob. Chang. Biol..

[14] Nybakken L, Julkunen‐Tiitto R (2013). Gender differences in Salix myrsinifolia at the pre‐reproductive stage are little affected by simulated climatic change. Physiol. plantarum.

[15] Baker J, Hartwell Allen L, Boote K, Pickering N (1997). Rice responses to drought under carbon dioxide enrichment. 1. Growth and yield. Glob. Chang. Biol..

[16] Houghton, J., Jenkins, G. & Ephraums, J. Climate change: the IPCC scientific assessment Cambridge University Press. *Cambridge, UK* (1990).

[17] Naumburg E, Loik ME, Smith SD (2004). Photosynthetic responses of *Larrea tridentata* to seasonal temperature extremes under elevated CO_2_. New Phytol..

[18] Paucar-Caceres A, Bandala ER, Wright GH (2017). The impact of global climate change on water quantity and quality: A system dynamics approach to the US–Mexican transborder regionAuthor-Name: Duran-Encalada, JA. Eur. J. Oper. Res..

[19] Vörösmarty CJ, Green P, Salisbury J, Lammers RB (2000). Global water resources: vulnerability from climate change and population growth. Sci..

[20] Webb RJ, McKellar R, Kay R (2013). Climate change adaptation in Australia: experience, challenges and capability development. Australas. J. Env. Man..

[21] Climate change in Australia. Technical Report. Available at, www.climatechangeinaustralia.gov.au (CSIRO, 2007)

[22] Ramesh K, Matloob A, Aslam F, Florentine SK, Chauhan BS (2017). Weeds in a changing climate: vulnerabilities, consequences, and implications for future weed management. Front. Plant Sci..

[23] Mehrtens J, Schulte M, Hurle K (2005). Unkrautflora in mais. Gesunde Pflanz..

[24] Otte A, Bissels S, Waldhardt R (2006). Samen-, Keimungs-und Habitateigenschaften: Welche Parameter erklären Veränderungstendenzen in der Häufigkeit von Ackerwildkräutern in Deutschland. J. Plant Dis. Protect.

[25] Chauhan BS, Johnson DE (2009). Seed germination ecology of junglerice (*Echinochloa colona*): a major weed of rice. Weed Sci..

[26] Ghannoum O (2008). C4 photosynthesis and water stress. Ann. Bot..

[27] Marenco Mendoza, R. & Lopes, N. F. *Fisiologia vegetal: fotossintese respiracao relacoes hidricas nutricao mineral*. (UFV, 2005).

[28] Larcher, W. *Physiological plant ecology: ecophysiology and stress physiology of functional groups*. (Springer Science & Business Media, 2003).

[29] Ohashi Y, Nakayama N, Saneoka H, Fujita K (2006). Effects of drought stress on photosynthetic gas exchange, chlorophyll fluorescence and stem diameter of soybean plants. Biol. Plant..

[30] Qaderi MM, Reid DM (2005). Growth and physiological responses of canola (*Brassica napus*) to UV‐B and CO_2_ under controlled environment conditions. Physiol. Plant..

[31] Qaderi MM, Kurepin LV, Reid DM (2006). Growth and physiological responses of canola (Brassica napus) to three components of global climate change: temperature, carbon dioxide and drought. Physiol. Plant..

[32] Pautasso M (2010). Plant health and global change–some implications for landscape management. Biol. Rev..

[33] Rosenzweig C (2014). Assessing agricultural risks of climate change in the 21st century in a global gridded crop model intercomparison. Proc. Natl. Acad. Sci..

[34] Holm, L. G., Plucknett, D. L., Pancho, J. V. & Herberger, J. P. *The world’s worst weeds. Distribution and biology*. (University press of Hawaii, 1977).

[35] Cameron, J. & Storrie, A. Summer fallow weed management. *Grains Research and Development Corporation GRDC* (2014).

[36] Widderick MJ, Bell KL, Boucher LR, Walker SR (2013). Control by glyphosate and its alternatives of glyphosate‐susceptible and glyphosate‐resistant *Echinochloa colona* in the fallow phase of crop rotations in subtropical Australia. Weed Biol. Manag..

[37] Maun MA, Barrett SCH (1986). The biology of Canadian weeds: 77. *Echinochloa crus-galli* (L.) Beauv. Can. J. Plant Sci..

[38] Chauhan BS, Johnson DE (2010). Growth and reproduction of junglerice (*Echinochloa colona*) in response to water stress. Weed Sci..

[39] Moss, S. R. Herbicide-resistant weeds. *Weed management handbook*. **9** (2002).

[40] Ahmadi MS, Haderlie LC, Wicks GA (1980). Effect of growth stage and water stress on barnyardgrass (*Echinochloa crus-galli*) control and on glyphosate absorption and translocation. Weed Sci..

[41] Tanpipat S, Adkins SW, Swarbrick JT, Boersma M (1997). Influence of selected environmental factors on glyphosate efficacy when applied to awnless barnyard grass (*Echinochloa colona* (L.) Link). Aus. J. Agric. Res..

[42] Mutti, N., Mahajan, G. & Chauhan, B. Seed germination ecology of glyphosate-resistant and glyphosate-susceptible biotypes of *Echinochloa colona* L.(Link) in Australia. *Weed Sci*. (accepted) (2019).

[43] Steadman KJ, Ellery AJ, Chapman R, Moore A, Turner NC (2004). Maturation temperature and rainfall influence seed dormancy characteristics of annual ryegrass (*Lolium rigidum*). Aust. J. Agric. Res..

[44] Geider, R. J. & Osborne, B. A. “Measuring Photosynthetic Pigments,” In *Algal Photosynthesis*, 107–121. (Springer, 1992).

[45] Hiscox JD, Israelstam GF (1979). A method for the extraction of chlorophyll from leaf tissue without maceration. Can. J. Bot..

[46] Kaspary TE, Lamego FP, Cutti L, Aguiar ACM, Bellé C (2014). Determination of photosynthetic pigments in fleabane biotypes susceptible and resistant to the herbicide glyphosate. Planta Daninha..

[47] Smillie RM, Nott R (1982). Salt tolerance in crop plants monitored by chlorophyll fluorescence *in vivo*. Plant Physiol..

[48] Clifford SC (2000). Effects of elevated CO_2_, drought and temperature on the water relations and gas exchange of groundnut (*Arachis hypogaea*) stands grown in controlled environment glasshouses. Physiol. Plant..

[49] Guy RD, Reid DM (1986). Photosynthesis and the influence of CO_2_‐enrichment on δ13C values in a C_3_ halophyte. Plant Cell Environ..

[50] Lawlor, D. W. & Mitchell, R. A. Crop ecosystem responses to climatic change: wheat. *Climate change and global crop productivity*, 57–80 (2000).

[51] Long SP, Ainsworth EA, Rogers A, Ort DR (2004). Rising atmospheric carbon dioxide: plants FACE the future. Ann. Rev. Plant Biol..

[52] Morrison, J. I. L. Stomatal response to increased CO_2_ concentration. J. *Exp. Bot*. (49), 443–452, www.jstor.org/stable/23695977 (1998).

[53] Taiz, L. & Zeiger, E. *Plant physiology*. 3rd edn. *Ann. Bot*. **91**, 750–751 (1991).

[54] Ziska LH, Bunce JA (1997). Influence of increasing carbon dioxide concentration on the photosynthetic and growth stimulation of selected C_4_ crops and weeds. Photosynth. Res..

[55] Knapp, A. K., Hamerlynck, E. P. & Owensby, C. E. Photosynthetic and water relations responses to elevated CO_2_ in the C_4_ grass Andropogon gerardii. *Inter. J. Plant Sci*. **154**, 459–466, www.jstor.org/stable/2995622 (1993).

[56] Broughton KJ (2017). Warming alters the positive impact of elevated CO_2_ concentration on cotton growth and physiology during soil water deficit. Funct. Plant Biol..

[57] Leakey AD (2009). Elevated CO_2_ effects on plant carbon, nitrogen, and water relations: six important lessons. J. Exp. Bot..

[58] Reddy AR, Chaitanya KV, Vivekanandan M (2004). Drought-induced responses of photosynthesis and antioxidant metabolism in higher plants. J. Plant Physiol..

[59] Drake BG, Gonzàlez-Meler MA, Long SP (1997). More efficient plants: a consequence of rising atmospheric CO_2_?. Annu. Rev. Plant Biol..

[60] Singh Rishi P., Prasad P. V. Vara, Reddy K. Raja (2013). Impacts of Changing Climate and Climate Variability on Seed Production and Seed Industry. Advances in Agronomy.

[61] Potvin C (1986). Biomass allocation and phenological differences among southern and northern populations of the C4 grass *Echinochloa crus-galli*. J. Ecol..

[62] Norris, R. F. Influence of soil moisture on activity of diclofop-methyl. In *Abstr. Weed Sci. Soc. Am*. 102–103 (1977).

[63] Merritt CR (1982). The influence of form of deposit on the phytotoxicity of MCPA, paraquat and glyphosate applied as individual drops. Ann. App. Biol..

[64] Neve P, Diggle AJ, Smith FP, Powles SB (2003). Simulating evolution of glyphosate resistance in *Lolium rigidum* II: past, present and future glyphosate use in Australian cropping. Weed Res..

[65] Wiese AF, Vandiver CW (1970). Soil moisture effects on competitive ability of weeds. Weed Sci..

